# FFPopSim: an efficient forward simulation package for the evolution of large populations

**DOI:** 10.1093/bioinformatics/bts633

**Published:** 2012-10-24

**Authors:** Fabio Zanini, Richard A. Neher

**Affiliations:** Evolutionary Dynamics and Biophysics Group, Max Planck Institute for Developmental Biology, 72076 Tübingen, Germany

## Abstract

**Motivation:** The analysis of the evolutionary dynamics of a population with many polymorphic loci is challenging, as a large number of possible genotypes needs to be tracked. In the absence of analytical solutions, forward computer simulations are an important tool in multi-locus population genetics. The run time of standard algorithms to simulate sexual populations increases as 8*^L^* with the number of loci *L*, or with the square of the population size *N*.

**Results:** We have developed algorithms to simulate large populations with arbitrary genetic maps, including multiple crossovers, with a run time that scales as 3*^L^*. If the number of crossovers is restricted to at most one, the run time is reduced to *L*2*^L^*. The algorithm is based on an analogue of the Fast Fourier Transform (FFT) and allows for arbitrary fitness functions (i.e. any epistasis). In addition, we include a streamlined individual-based framework. The library is implemented as a collection of C++ classes and a Python interface.

**Availability:** http://code.google.com/p/ffpopsim/.

**Contact:** richard.neher@tuebingen.mpg.de

**Supplementary information:** Supplementary data are available at *Bioinformatics* online.

## 1 INTRODUCTION

**Fig. 1.**
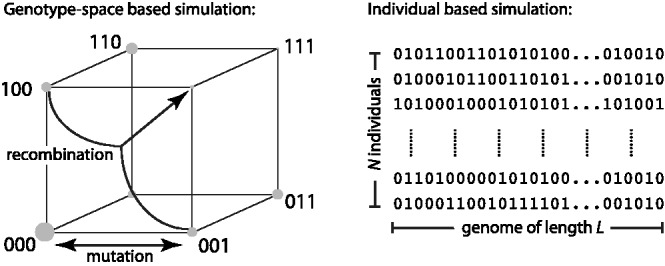
Strategies for forward simulations: The left panel illustrates a scheme that tracks the abundance of each possible genotype, encoded as a bit string. This is feasible up to *L* ≈ 20 and is implemented in FFPopSim as the class haploid_lowd. Recombination requires considering all possible pairs of parental genotypes and the different ways their genomes can be combined, which is computationally expensive. The right panel illustrates individual-based simulations that track existing genotypes only. FFPopSim provides individual-based simulations through the class haploid_highd

Forward simulations of population genetics track either the number of individuals carrying each possible genotype or the genotype of every individual in the population, as illustrated in Figure 1. The latter strategy has been implemented in a number of flexible simulation packages (Guillaume and Rougemont, 2006; Peng and Kimmel, 2005; Spencer and Coop, 2004). In large populations with a moderate number of loci *L*, storing the abundance of all possible 2*^L^* genotypes is often faster. Simulating such large populations with a small number of loci is for example essential when studying the evolution of drug resistance in viral or bacterial pathogens.

Individual-based population genetics simulations are straightforward and usually use a discrete generation scheme in which processes such as mutation, selection and migration are applied at every generation to every individual. Individuals are then paired up via a mating scheme and recombinant offspring are produced. Existing toolboxes often emphasize biological realism and allow the user to specify complex life cycles. Our emphasis here is on efficient simulation of large populations. The class haploid_lowd of FFPopSim tracks the distribution *P*(*g*) of gametes across all possible 2*^L^* genotypes, denoted by 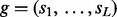 where *s_i_* = 0/1 (see Fig. 1). This genotype distribution changes owing to mutation, selection and recombination. In our implementation, selection acts on haploid gametes, precluding dominance effects. Recombination is a computationally expensive operation, as it involves pairs of parents (up to 4*^L^* of them) that can combine their genome in many different ways (2*^L^* for arbitrary genetic maps, 2*L* when allowing single crossovers only). As a consequence, a naive implementation requires 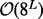 or 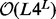 operations to calculate the distribution of recombinant genotypes for arbitrary or single crossover recombination, respectively. It is intuitive that the complexity of this algorithm can be reduced: given a recombination pattern, only a fraction of the genome is passed on and all genotypes that agree on that fraction contribute identically.

We show in the Supplementary Material that the distribution of recombinants can be calculated in 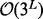 steps for arbitrary crossover patterns and in 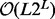 steps if only a single crossover is allowed. This reduces the complexity of evolving sexual populations to that of asexual populations. The crucial ingredient for the increased performance is a Fast Fourier Transform on genotype spaces explained in detail in the Supplementary Material. The genotype space is an *L* dimensional hypercube on which Fourier and Taylor expansions can be defined.

After selection, mutation and recombination, the population distribution *P*(*g*) contains the expected number of individuals of genotype *g* in the next generation. For stochastic population genetics, we still need to resample the population in a way that mimics the randomness of reproduction. This is achieved by resampling individuals according to a Poisson distribution with mean *N P*(*g*) for each genotype. The resulting population has a size of approximately 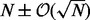. The fluctuations in the population size are small if *N* is large and do not propagate since the population is resampled *de novo* each generation. The user can set the expected population size before resampling and generate any desired time-dependent population size. For a general discussion of multi-locus evolution see e.g. (Neher and Shraiman, 2011).

**Fig. 2.**
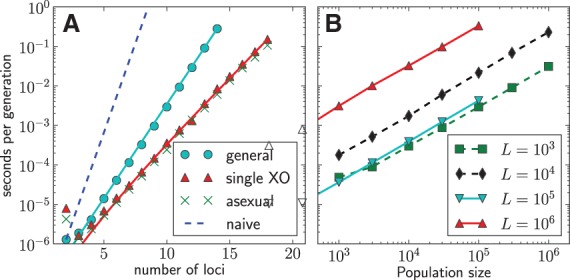
Performance of FFPopSim. (**A**) The time required to simulate a single generation as a function of the number of loci, using the class haploid_lowd. The expected scalings [8*^L^* for naive implementation, 3*^L^* for general recombination and *L*2*^L^* for single crossovers (XO)] are indicated by solid lines. (**B**) The run times of the individual-based simulations as a function of the population size for different genome sizes *L* using haploid_highd. Solid lines correspond to crossover and mutations rates *ρ* = *μ* = 10^−8^ typical of the human genome, dashed lines to outcrossing with rate *r* = 0.01, and *μ* = 10^−5^, *ρ* = 10^−3^ typical for viral evolution. Run times were determined on a 2.93 GHz Intel CPU

Figure 2 shows how the time required to simulate one generation increases with the number of loci, confirming the expected scaling behaviour of the computational complexity.

## 2 USAGE

FFPopSim is implemented in C++ with a Python2 wrapper. From Python, FFPopSim can be imported as a module. We provide examples that reproduce basic population genetics results, such as a mutation–selection–drift equilibrium or the decay of linkage disequilibrium. The C++ library can be linked against custom code and modified for specific purposes. The class itself is called haploid_lowd, as it simulates a haploid population with a relatively small number of loci.

As an example, we discuss here the problem of fitness valley crossing, which has recently received attention in the population genetics literature (Lynch, 2010; Weissman *et al.*, 2010) and requires forward simulation of large populations while tracking the state of several loci. Consider a fitness landscape where the wild-type genotype has (Malthusian) fitness *s*_1_, the quadruple mutant has fitness *s*_1_ + *s*_2_, and all intermediate genotypes have the same slightly deleterious fitness 0 (−*s*_1_ relative to wild type). The time required for crossing the valley can be computed by the following routine:

import FFPopSim

L = 4     # number of loci

N = 1e10    # population size

# create population and set rates

p = FFPopSim.haploid_lowd(L)

p.set_recombination_rates(0.001)

p.set_mutation_rates(1e-5)

# start with wildtype: 0b0000 = 0

p.set_genotypes ([0b0000], [N])

# set positive relative fitness for wildtype

# and quadruple mutant: 0b1111 = 15

p.set_fitness_function ([0b0000, 0b1111],              [0.01, 0.02])

# evolve until the quadruple mutant spreads

while p.get_genotype_frequency(0b1111) < 0.5: p.evolve(100)

print p.generation

The run time and memory requirements of 3*^L^* or *L*2*^L^* still preclude the simulation of more than *L* = 20 loci. For this reason, we also include the class haploid_highd to perform individual-based simulations with the same interface. haploid_highd can simulate an arbitrarily large number of loci and has overall run time and memory requirements 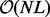 in the worst case scenario (see Fig. 2B). Identical genotypes are grouped into clones to speed up the simulation whenever diversification via mutation or recombination is rare (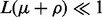, where *μ* and *ρ* are mutation and recombination rates, respectively). The population size can be adjusted at any time by changing the carrying capacity. Overall, haploid_highd can simulate one megabase genomes in a population of 10^5^ individuals in about one second per generation on a present day desktop computer. We have written a specific subclass hivpopulation for large viral populations that is also included in FFPopSim. As of now, the library does not support dominance effects; that would require a fitness function that depends on pairs of haploid genomes. Such an extension to diploid populations is planned.

## Supplementary Material

Supplementary Data

## References

[bts633-B1] Guillaume F., Rougemont J. (2006). Nemo: an evolutionary and population genetics programming framework. Bioinformatics.

[bts633-B2] Lynch M. (2010). Scaling expectations for the time to establishment of complex adaptations. Proc. Natl Acad. Sci. USA.

[bts633-B3] Neher R., Shraiman B. (2011). Statistical genetics and evolution of quantitative traits. Rev. Mod. Phys..

[bts633-B4] Peng B., Kimmel M. (2005). simupop: a forward-time population genetics simulation environment. Bioinformatics.

[bts633-B5] Spencer C.C.A., Coop G. (2004). Selsim: a program to simulate population genetic data with natural selection and recombination. Bioinformatics.

[bts633-B6] Weissman D. (2010). The rate of fitness-valley crossing in sexual populations. Genetics.

