# Supplementary material for: FFPopSim: an efficient forward simulation package for the evolution of large populations
Source: Bioinformatics. 2012 Oct 24;28(24):3332–3. doi: 10.1093/bioinformatics/bts633 (PMC3519462; doi:10.1093/bioinformatics/bts633)
Supplement: Supplementary Data [file supp_28_24_3332__index.html]

FFPopSim: an efficient forward simulation package for the evolution of large populations — Supplementary Data 

# FFPopSim: an efficient forward simulation package for the evolution of large populations

## Supplementary Data

files

**Files in this Data Supplement:**

- Supplementary Data - pdf file
