# Supplementary material for: FFPopSim: an efficient forward simulation package for the evolution of large populations
Source: Bioinformatics. 2012 Oct 24;28(24):3332–3. doi: 10.1093/bioinformatics/bts633 (PMC3519462; doi:10.1093/bioinformatics/bts633)
Supplement: Supplementary Data [file supp_bts633_FFPopSimDocumentation_revision.pdf]

FFPopSim: An efficient forward simulation package for the evolution of large populations

Fabio Zanini and Richard A. Neher

Max Planck Institute for Developmental Biology, 72076 Tübingen, Germany

(Dated: September 26, 2012)

FFPopSim is toolbox for forward simulations in population genetics. It is coded in C++. For easy and interactive use, a Python interface is provided such that FFPopSim can be loaded as a module in Python. A comprehensive documentation of the C++ and Python parts of the library can be found on the FFPopSim webpage:

`http://webdav.tuebingen.mpg.de/ffpopsim`

The following pages explain the algorithms underlying the library and provide comprehensive tables listing all methods and members available through the python interface.

Contents

I. Prerequisites and compiling 1

II. Conventions and definitions 2

III. Description of FFPopSim 2
- A. Fast-Fourier Transform of functions of the genotype 3
- B. FFPopSim for few loci: `haploid_lowd` 3
  - 1. Specification of the population and the fitness function 3
  - 2. Evolution 4
  - 3. Mutation 4
  - 4. Selection 5
  - 5. Resampling and genetic drift 5
  - 6. Mating and recombination 5
- C. FFPopSim for many loci: `haploid_highd` 7
  - 1. Specification of the population and the fitness function 7
  - 2. Mutation 7
  - 3. Selection 8
  - 4. Recombination 8
- D. Python wrapper 8

IV. Python functions 9
- A. `haploid_lowd` 9
- B. `haploid_highd` 10
- C. `hivpopulation` 11

V. Examples 12
- A. `haploid_lowd`: Valley crossing 12
- B. `haploid_lowd`: HIV immune escape 14
- C. `haploid_highd`: Genetic draft and drift 14
- D. `hivpopulation`: HIV chronic infection 17

References 17

I. PREREQUISITES AND COMPILING

We provide binaries of the python library for recent Linux or MacOS versions (see online documentation). Those can be installed without any compilation and only require and up-to-date Python distribution, including NumPy, SciPy and MatplotLib (`http://www.scipy.org`).

To compile the library from the C++ source, you need the GNU scientific library (`http://www.gnu.org/software/gsl/`) and the Boost C++ library (`http://www.boost.org/`). If all of these are installed and the appropriate path are set, FFPopSim can be compiled using Make. Make will compile the C++ code and call `setup.py`, which uses python `distutils` to make sure the correct python path are used. Installation instructions are provided in the `INSTALL` file. The building process creates files inside the folder `pkg`; C++ headers are created in `pkg/include`, the static C++ library in `pkg/lib`, and the Python module files in `pkg/python`.

The python interface to the C++ library is produced using `SWIG`. The output files of SWIG are platform independent and included in the source. Hence `SWIG` is required only if the source is changed and the python binding need to be rebuilt. For this reason, `make clean` will not delete `SWIG` output. Similarly, the documentation is produced using `Doxygen` (the C++ part) and `Sphinx` (the python part) and shipped with the source. `make clean-all` deletes documentation and `SWIG` output.

II. CONVENTIONS AND DEFINITIONS

`FFPopSim` assumes **haploid** individuals. This imposes no restriction as long as dominance effects are not important. An extension to diploids is envisioned. Any population size we refer to is therefore the number of haploid genomes in the populations.

`FFPopSim` is strictly **biallelic**: Each locus can be in one of two states, conveniently stored as one bit 0/1.

A population in `FFPopSim` is endowed with a genotype to fitness map. There are many ways in which such a map can be specified. Most straightforwardly, one explicitly assignes a fitness value to each genotype. However, there are prohibitively many genotypes to store and specify if the number of loci is large and this option is only supported in `haploid_lowd`. With biallelic loci, there are 2^L possible genotypes and the most general function would need to be specified for each of these.

Alternatively, a genotype-phenotype map can be specified relative to a reference genotype, commonly referred to as wild type. If the wild type genome is $g_{WT} = \{0, 0, \ldots, 0\}$, the fitness or phenotype of any other genome $g = \{s_1, \ldots, s_L\}$ is then

$$F(g) = F_{WT} + \sum_i s_i c_i^{(1)} + \sum_{i>j} s_i s_j c_{ij}^{(2)} + \ldots \tag{1}$$

where $c_i^{(1)}$ is the effect of a substitution at locus i, while $c_{ij}^{(2)}$ is the epistatic effect of a double substitution at loci i and j. For computational reasons that will become clear below, it is useful to reformulate this map in a basis where a locus takes the states $t_i \in \{-1, +1\}$ rather than $s_i \in \{0, 1\}$. The $\{-1, +1\}$ basis is much more symmetric and this symmetry can be used to speed up calculations. For this reason `FFPopSim` uses the $\{-1, +1\}$ basis. Since most population geneticists are more familiar with the $\{0, 1\}$ basis, we discuss their relation here.

The two bases are related to each other by a simple linear transformation

$$t_i = 2s_i - 1 \quad \text{and its inverse} \quad s_i = \frac{1}{2}(t_i + 1) \tag{2}$$

In this symmetric basis, a genotype phenotype map is specified as

$$F(g) = f^{(0)} + \sum_i t_i f_i^{(1)} + \sum_{i<j} t_i t_j f_{ij}^{(2)} + \cdots \tag{3}$$

The coefficients of this expansion $f_i^{(k)}$ are related to the $c_i^{(k)}$ as follows:

$$f_i^{(1)} = \frac{1}{2} c_i^{(1)} + \frac{1}{4} \sum_j c_{ij}^{(2)} + \ldots \quad \text{and} \quad f_{ij}^{(1)} = \frac{1}{4} c_{ij}^{(2)} + \ldots \tag{4}$$

Hence, for most practical purposes the single locus selection coefficients in the $\{-1, +1\}$ basis are simply half of the single locus selection coefficients in $\{0, 1\}$ basis. Similarly, the two locus interaction coefficients $f_{ij}^{(2)}$ are simply a quarter of their counter part $c_{ij}^{(2)}$. Note, however, that interactions in the $\{0, 1\}$ bases contribute to the single locus coefficents in the $\{-1, +1\}$ basis. The constant term $f^{(0)}$ is rarely important since all fitness values are relative to a mean fitness.

FFPopSim stores all fitness values as log-fitness. Conventional fitness is then obtained by exponentiation: $W(g) = \exp(F(g))$. Specification of an additive fitness landscape in terms of $F(g)$ results in multiplicative fitness in terms of $W(g)$.

III. DESCRIPTION OF FFPOPSIM

`FFPopSim` contains two packages of C++ classes and Python wrappers for forward population genetics simulations. One for large populations and relatively few loci ($L < 20$), another one for longer genomes. The former is called `hapoid_lowd` and

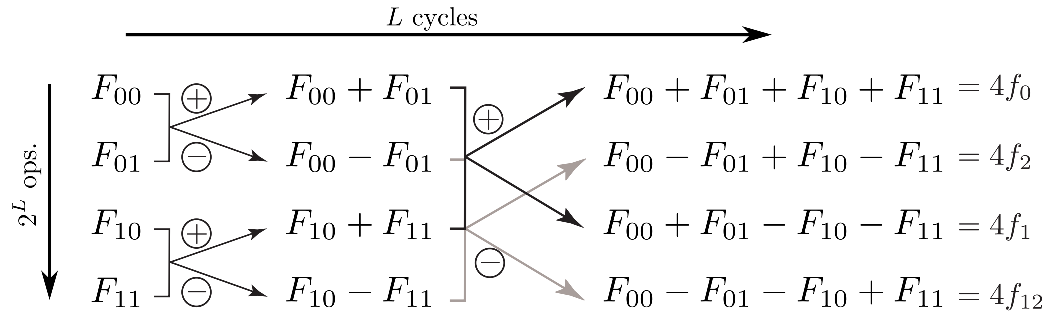

Figure 1 Calculating the Fourier transform in $L2^L$ operations. Arrow going up indicate addition, going down substraction. For the general L dimensional hypercubes L cycles are necessary where terms differing at different bits are combined.

tracks the abundance of all possible genotypes. The other one is called `haploid_highd`, tracks only genotypes present in the population, and is essentially individual-based.

`haploid_highd` only allows for limited complextity of fitness functions and crossover patterns, while `hapoid_lowd` supports arbitrarily complex fitness functions and genetic maps. The two parts of the library have very similar syntax but work quite differently under the hood. We will therefore describe them separately below.

A complete `html` documentation of the C++ functions is generated automatically from the source using Doxygen and can be found in `pkg/doc/cpp/html/index.html`. All python functions are documented using Sphinx and can be found in `pkg/doc/python/html/index.html`. The following is a rather technical description of the algorithms used in the library and how they are implemented as C++ functions. To simply get an impression of what can be done with `FFPopSim`, the reader can fast-forward to the examples in section V.

A. Fast-Fourier Transform of functions of the genotype

Above, we introduced a specification of genotype to phenotype functions by building them from single locus effects, pair-wise interaction terms, and so forth. In total there are 2^L such coefficients – enough to specify the most general function. Any function $F(g)$ on the hypercube can be expressed as

$$F(g) = f^{(0)} + \sum_i t_i f_i^{(1)} + \sum_{i<j} t_i t_j f_{ij}^{(2)} + \cdots \qquad (5)$$

There are $\binom{L}{k}$ coefficients $f^{(k)}_{i_1 \dots i_k}$ for every subset of k loci out of L loci. There are 2^L coefficients in total (Weinberger, 1991). A coefficient $f^{(k)}_{i_1 \dots i_k}$ is uniquely specified by

$$f^{(k)}_{i_1 \dots i_k} = 2^{-L} \sum_g t_{i_1} \dots t_{i_k} F(g) \qquad (6)$$

These nominally 4^L operations (2^L for each coefficient) can be done in $L2^L$ steps via the Fast Fourier Transform (FFT) illustrated in Fig. 1. Both the forward and reverse transform are implemented in `hypercube_lowd`.

This FFT is central to the efficient simulation of large populations and used extensively across `haploid_lowd`.

B. FFPopSim for few loci: `haploid_lowd`

A complete list of all function with descriptions, call and caller graphs, is generated automatically from the source in `html` by Doxygen. Here, we describe the most important functions and describe the recombination algorithm.

1. Specification of the population and the fitness function

An instance of `hypercube_lowd` can be initialized with the function values of the hypercube or with its Fourier coefficients. The population class, `haploid_lowd`, holds instances of `hypercube_lowd` for the population, the mutant genotypes, the recombinant genotypes and the fitness function.

From a practical point of view, an instance of a low-dimensional population is initialized in three steps,

(a) the class is instantiated

```
haploid_lowd::haploid_lowd(int L=1, int rng_seed=0)
```

where L is the number of loci and `rng_seed` is a seed for the random number generator (a random seed by default);

(b) the initial population can be specified by one of the functions

```
int haploid_lowd::set_allele_frequencies(double *freq, unsigned long N)
int haploid_lowd::set_genotypes(vector <index_value_pair_t> gt)
int haploid_lowd::set_wildtype(unsigned long N)
```

that set the population size and composition. The first function initializes random genotypes in linkage equilibrium with the specifiec allele frequencies `freq`, the second explicitely sets a number of individuals for each genotype using the new type `index_value_pair`, and the last one sets a wild type-only (genotype 000...00) population of size `N`;

(c) the fitness function is stored in `haploid_lowd` in an instance of `hypercube_lowd` which is a public member `haploid_lowd.fitness`. To inialize, the following functions are available:

```
int hypercube_lowd::additive(double* additive_effects, bool add)
int hypercube_lowd::init_list(vector <index_value_pair_t> iv, bool add)
int hypercube_lowd::init_coeff_list(vector <index_value_pair_t> iv, bool add)
```

The `hypercube_lowd::additive` and `hypercube_lowd::init_coeff_list` allow to set either all additive coefficients, or a vector of `index_value_pair` where the index specifies the interaction term (00101 would be a interaction between locus 0 and 2). `hypercube_lowd::init_list` specifies fitness of genotypes directly, rather than coefficients. The index in `index_value_pair` then specifies the genotype again in binary format.

2. Evolution

In traditional Wright-Fisher type models, in each generation, the expected frequencies of gametes with a particular genotype after mutation, selection, and recombination are calculated and then the population resampled from this gamete distribution. We will now outline the steps required to update the population. A more detailed discussion can be found in (Neher and Shraiman, 2011). All the following steps are called by either one of the following functions:

- `int haploid_lowd::evolve(int gen=1)`

  updates the population looping over a specified number of generations `gen`;

- `int haploid_lowd::evolve_deterministic(int gen=1)`

  is an alternative version that skips the resampling (deterministic evolution);

- `int haploid_lowd::evolve_norec(int gen=1)`

  is another alternative that skips the recombination step (asexual evolution).

3. Mutation

Let $P(g)$ be the genotype distribution at the beginning of a generation. Denoting the mutation rate towards the 1 or 0 state at locus i by $u_i^{1/0}$, the expected $P(g)$ of genotype $g = \{s_1, \ldots s_L\}$ after mutation will be

$$P(g) \quad \longleftarrow \quad \left(1 - \sum_{i=0}^{L-1} u_i^{\overline{s_i}}\right) P(g) + \sum_{i=0}^{L-1} u_i^{s_i} P(\Pi_i g) \tag{7}$$

where $\Pi_i g$ denotes genotype g with locus i flipped from 0 to 1 or vice versa. The first term is the loss due to mutation, while the second term is the gain due to mutation from neighbouring genotypes (in terms of Hamming distance).

The mutation rates can be specified by the folowing set of overloaded functions: either a single double rate (same for every position and in both forward and backward sense), two double rates (forward and backward rates), a L dimensional double array (site-specific, identical forward and backward rates), or a $2 \times L$ dimensional double array,

- `int haploid_lowd::set_mutation_rate(double rate);`

  takes a single rate and sets it for every position and in both forward and backward direction;

- `int haploid_lowd::set_mutation_rate(double rate_forward, double rate_backward);`

  takes two rates and sets `rate_forward` as the forward rate ($0 \to 1$) and `rate_backward` as the backward rate ($1 \to 0$)

- `int haploid_lowd::set_mutation_rate(double* rates);`

  takes the pointer to an array of length L and sets site-specific rates, the same for forward and backward mutations;

- `int haploid_lowd::set_mutation_rate(double **rates)`

  takes a pointer to a pair of (pointers to) arrays, each of length L, which contain the site-specific rates for forward (`rates[0]`) and backward (`rates[1]`) mutations.

4. Selection

Selection reweighs different genotypes according to their fitness as follows

$$P(g) \quad \longleftarrow \quad \frac{e^{F(g)}}{\langle e^F \rangle} P(g) \tag{8}$$

where $\langle e^F \rangle$ is the population average of $e^{F(g)}$, which is required to keep the population size constant. The corresponding function is

```
int haploid_lowd::select_gametes()
```

5. Resampling and genetic drift

For deterministic modeling, one generation would be completed at this point and one would repeat the cycle, starting with mutation again. For stochastic population genetics, we still need to resample the population in a way that mimics the randomness of reproduction. The easiest and most generic way to do this is to resample a population of size N using a multinomial distribution with the current $P(g)$ as sampling probabilities of different genotypes. Alternatively, one can sample individuals according to a Poisson distribution with mean $NP(g)$ for each genotype, which will result in a population of size $N \pm O(\sqrt{N})$. For large populations, the two ways of resampling are equivalent and we chose the latter (much faster) alternative. The function

```
int haploid_lowd::resample()
```

samples the next generation according to the expected genotype frequencies. The expected population size used in the resampling is the carrying capacity, which can be set by the user at will.

6. Mating and recombination

The computationally expensive part of the dynamics is recombination, which needs to consider all possible pairs of pairs of parents and all different ways in which their genetic information can be combined. In a facultatively sexual population, a fraction r of the individuals undergo mating and recombination. In obligate sexual populations, $r = 1$. The genotype distribution is updated according to the following rule:

$$P(g) \quad \longleftarrow \quad (1-r)P(g) + rR(g) \tag{9}$$

The distribution $R(g)$ of recombinant gametes would naively be computed as follows:

$$R(g) = \sum_{\xi} \sum_{g'} C(\xi) P(g^m) P(g^p), \tag{10}$$

where ξ specifies the particular way the parental genomes are combined: $\xi_i = 0$ (resp. 1) if locus i is derived from the mother (resp. father). The genotype g' is summed over; it represents the part of the maternal (g^m) and paternal (g^p) genotypes that is not

passed on to the offspring. We can decompose each parent into successful loci that made it into the offspring and wasted loci, as follows: $g^p = \xi \wedge g + \overline{\xi} \wedge g'$ and $g^m = \overline{\xi} \wedge g + \xi \wedge g'$, where $\wedge$ and a bar over a variable indicate respectively the elementwise AND and NOT operators (i.e., $\overline{\xi_i} := 1 - \xi_i$). The function C assigns a probability to each inheritance pattern. Depending on whether the entire population undergoes sexual reproduction or only a fraction r of it, the entire population or a fraction r is replaced with $R(g)$. The central ingredient for the efficient computation of $R(g)$ is the Fourier decomposition introduced above. The generic Fourier coefficient of $R(g)$ is given by

$$r^{(k)}_{i_1 \dots i_k} = 2^{-L} \sum_g t_{i_1} \dots t_{i_k} \left(\sum_\xi \sum_{g'} C(\xi) P(g^m) P(g^p) \right) \tag{11}$$

Just as g^p and g^m can be expressed as a combination of g and g', we can invert the relation and express the generic t_i as a function of g^p and g^m, as follows: $t_i = \xi_i t^m_i + \overline{\xi_i} t^p_i$. Using this new basis and exchanging the order of summations, we obtain

$$r^{(k)}_{i_1 \dots i_k} = 2^{-L} \sum_\xi C(\xi) \sum_{g^m, g^p} (\xi_{i_1} t^m_{i_1} + \overline{\xi_{i_1}} t^p_{i_1}) \dots (\xi_{i_k} t^m_{i_k} + \overline{\xi_{i_k}} t^p_{i_k}) P(g^m) P(g^p). \tag{12}$$

Notice that $C(\xi)$ can be pulled out of the two inner sums, because the odds of inheriting a certain locus by the mother/father is independent of what their genetic makeup looks like. Next we expand the product and introduce new labels for compactness,

$$r^{(k)}_{i_1 \dots i_k} = 2^{-L} \sum_\xi C(\xi) \sum_{g^m, g^p} P(g^m) P(g^p) \sum_{l=0}^{k} \sum_{\{j_i\},\{h_i\}} \xi_{j_1} \dots \xi_{j_l} \overline{\xi_{h_1}} \dots \overline{\xi_{h_{k-l}}} t^m_{j_1} \dots t^m_{j_l} t^p_{h_1} \dots t^p_{h_{k-l}}, \tag{13}$$

where l is the number of loci inherited from the mother among the k in $(i_1, \dots, i_k)$. l runs from 0 (everything is contributed by the father) to k (everything from the mother). $\{j_i\}$ and $\{h_i\}$ are all (unordered) partitions of i into sets of size l and $k-l$, respectively. Now we can group all ξ_i in the inner sum with $C(\xi)$, all t^m_i with $P(g^m)$, and all t^p_i with $P(g^p)$. The three sums (over ξ, g^m, and g^p) are now completely decoupled. Moreover, the two sums over the parental genotypes happen to be the Fourier decomposition of $P(g)$. Hence, we have

$$r^{(k)}_{i_1 \dots i_k} = \sum_{l=0}^{k} \sum_{\{j_i\},\{h_i\}} C^{(k)}_{j_1 \dots j_l, h_1 \dots h_{k-l}} p^{(k)}_{j_1 \dots j_l} p^{(k-l)}_{h_1 \dots h_{k-l}}. \tag{14}$$

The quantity

$$C^{(k)}_{j_1 \dots j_l, h_1 \dots h_{k-l}} = \sum_\xi C(\xi) \xi_{j_1} \dots \xi_{j_l} \overline{\xi_{h_1}} \dots \overline{\xi_{h_{k-l}}} \tag{15}$$

can be calculated efficiently, for each pair of partitions $(\{j_i\}, \{h_i\})$, by realizing that (a) for $k = L$, there is exactly one term in the sum on the right that is non-zero and (b) all lower-order terms can be calculated by successive marginalizations over unobserved loci. For instance, let us assume that $k = L-1$ and that the only missing locus is the m-th one. We can compute

$$C^{(L-1)}_{j_1 \dots j_l, h_1 \dots h_{L-1-l}} = C^{(L)}_{j_1 \dots j_l m, h_1 \dots h_{L-1-l}} + C^{(L)}_{j_1 \dots j_l, h_1 \dots h_{L-1-l} m}. \tag{16}$$

There are $\binom{L}{k}$ ways of choosing k loci out of L, which can be inherited in 2^k different ways (the partitions in j and h in Eq. (15)) such that the total number of coefficients is 3^L. Note that these coefficients are only calculated when the recombination rates change. Furthermore, this can be done for completely arbitrary recombination patterns, not necessarily only those with independent recombination events at different loci.

The recombination process is greatly simplified if only one crossover is allowed. In this case, the majority of the coefficients $C^{(L)}_{j_1 \dots j_l m, h_1 \dots h_{L-1-l}}$ are zero. In fact, there are only L allowed recombination patters and the sum over the different partitions in Eq. (15) has only $k+1$ allowed terms. In this case, the entire recombinant distribution can be calculated in $O(L2^L)$ steps and is therefore of the same complexity as the mutation step.

`haploid_lowd` provides a function to calculate $C(\xi)$ from recombination rates between loci assuming a circular or linear chromosome. The probability of a particular crossover pattern is calculated assuming independent crossovers. The function

```
haploid_lowd::set_recombination_rates(double *rec_rates, int rec_model)
```

assumes a double array of length $L-1$ for a linear chromosome and of length L for a circular chromosome. For a linear (resp. circular) chromosome, the i-th element of the array is the probability of recombining after (resp. before) the i-th locus. The recombination model is specified as an optional argument and can take values `CROSSOVERS`, `SINGLE_CROSSOVER` and

FREE_RECOMBINATION specifed in the header file. If the recombination model is CROSSOVERS, independent crossovers between loci are assumed and multiple crossovers allowed. SINGLE_CROSSOVER restricts to at most one crossover (not available for circular genomes), and the recombination rates are interpreted as probabilities of observing a crossover between two loci (their sum needs to be less than one). haploid_lowd offers the simpler alternative for free recombination which reassorts all loci at random. If the user does not set the recombination rates via set_recombination_rate, free recombination is the default behaviour.

Other more complicated recombination patterns can be specified manually through

```
haploid_lowd::set_recombination_patterns(vector<index_value_pair_t> iv)
```

which takes a vector of patterns and the associated rates. All other rates are assumed 0. Remember to specify the rate at which no recombination happens (pattern 000000 or 111111).

Furthermore, the mating probability r can be specified explicitely via the attribute

```
haploid_lowd::outcrossing_rate
```

the default is obligate sexual reproduction, i.e., outcrossing_rate=1.

Note that, in a circular chromosome, there is effectively one more inter-locus segment (between the last and the first locus) in which crossovers can occur, and the total number of crossovers has to be even. Assuming independent crossovers, the global recombination rate of circular chromosomes is lower than a linear chromosome of the same length by a factor of $(1-e^{-r_0})$, where r_0 is the recombination rate between the first and last loci.

The recombination process itself is initiated by

```
int haploid_lowd::recombine()
```

C. FFPopSim for many loci: haploid_highd

For more than 20 loci, storing then entire genotype space and all possible recombination patterns becomes prohibitive. For this reason, we also include a streamlined individual-based simulation package that can simulate arbitrarily large numbers of loci and has an overall run-time and memory requirements $O(NL)$ in the worst case scenario. The many-loci package uses the same interface as the few-loci one. This makes it easy, for example, to first test an evolutionary scenario using many (all) loci and to focus on the few crucial ones afterwards.

The one notable difference is that fitness in haploid_highd is a function of several traits, all of which are functions on the hypercube. This is useful, for example, to model changing treatment with antiviral drugs: fitness is then a function of replicative capacity and drug resistance. The mapping of traits to fitness can be changed by defining the function

```
void calc_individual_fitness_from_traits(clone_t *tempgt);
```

which is a virtual member of haploid_highd.

To speed up the program in many cases of interest, identical genotypes are grouped into clones. The way the population is initialized and evolution is controlled is largely identical to hypercube_lowd above. We only highlight the differences here. A full html documentation and many examples are provided with the source.

1. Specification of the population and the fitness function

haploid_highd stores individual genotypes as bitsets. Each genotype, together with the number of individuals that carry it, as well as traits and fitness associated with it is stored for as long as it is present in the population. All relevant quantities are aggregated in the structure clone_t. The population is a vector of clones. Each generation clone sizes are updated, new clones that arise through mutation and recombination are added, and clones of size 0 marked for future reuse.

Traits are functions on the hypercube. The latter is implemented as hypercube_highd. Instead of storing all possible trait values, hypercube_highd stores non-zero Fourier coefficients. Whenever a new genotype is produced, its fitness is calculated by summing the appropriate coefficients. No explicit specification of trait values for particular genotypes is possible.

Fitness is a function of the traits of individuals. By default, fitness is identical to the value of the first trait. The mapping from traits to fitness is defined as a virtual function and can be overwritten by the inheriting classes.

2. Mutation

To implement mutation, we determine the individuals that mutate (each indiviudal has probability of $1-\exp(-L\mu)$ to mutate). In each of these individuals, at least one, and possible several mutations are introduced in random locations in the genome. Mutations are bit-flip operations in the bitset. As of now, mutation rates are the same at every locus in the genome.

3. Selection

Prior to selection, the population average $\overline{W} := \langle e^F \rangle$ and a growth rate adjustment $\exp(1-N/N_0)$ are computed. The latter is used to keep the population size close to the carrying capacity N_0. The size of each clone is then updated with a Poisson distributed number with mean $(1-r)\overline{W}^{-1}\exp(F(g)+1-N/N_0)$, where r is the probability of outcrossing (only non-outcrossing individuals remain part of the same clones). The number of individuals that outcross is again Poisson distributed number with mean $r\overline{W}^{-1}\exp(F(g)+1-N/N_0)$. Those individuals are saved for recombination in the next step.

4. Recombination

The individuals marked for sexual reproduction during the selection step are shuffled and paired. For each pair, a bitset representing the crossover pattern is produced and two new clones are produced from the two parental genomes. Crossovers are assumed to occur independently between any two loci with the crossover rate. Alternatively, all loci of the two genomes can be reassorted at random. The two allowed values for `recombination_model` are `FREE_RECOMBINATION` and `CROSSOVERS`.

D. Python wrapper

The C++ library includes Python bindings that greatly simplify interactive use and testing. The wrapping itself is done by SWIG (Beazley, 2003). Most notably, the C++ classes `haploid_lowd`, `haploid_highd` and the HIV-specific subclass `hiv_population` are fully exposed to Python, including all their public members. The performance speed for evolving a population is unchanged, since the `evolve` function iterates all steps internally for an arbitary number of generations.

The bindings are not completely faithful to the C++ interface, to ensure a more intuitive user experience. For instance, some C++ attribute set/get members are translated into Python properties via the builtin `property` construct. Furthermore, since direct access to the `hypercube_lowd` instances from Python is not straightforward, a few utility functions have been written to do common tasks. The fitness hypercube can be set easily by either one of

```
haploid_lowd.set_fitness_function(genotypes, fitnesses)
haploid_lowd.set_fitness_additive(selection_coefficients)
```

The former function is used to set specific points on the hypercube: it takes a sequence of genotypes `genotypes` (integers or binary literals using `0b` notation) and a sequence of fitness values `fitnesses`, corresponding to those genotypes. Fitness values are interpreted as growth rates, also known as log-fitnesses. Any missing points on the fitness hypercube will be set to $F(g) = 0$. The second function creates an additive fitness landscape with selection coefficients specified by the L-dimensional input sequence `selection_coefficients`, see Eq. (3). After installation, the FFPopSim library can be used in Python as a module, e.g.

```
from FFPopSim import haploid_lowd
```

The bindings make heavy use of the NumPy library and its SWIG fragments and typemaps (Oliphant, 2006). We therefore recommend to import NumPy before FFPopSim, although this is not strictly necessary. Moreover, the Python bindings include a few functions for plotting features of the population, such as genetic diversity. The Python module Matplotlib is required for this purpose (Hunter, 2007).

The HIV-specific part of the code has been expanded further in Python to enable quick simulations of viral evolution under commonly studied conditions. In particular, random genotype-phenotype maps for viral replication capacity and drug resistance can be generated automatically from a few parameters, via the functions

```
hivpopulation.set_replication_landscape
hivpopulation.set_resistance_landscape
```

Both functions have many input parameters that allow to specify typical properties of HIV populations, such as the fraction of sites carrying potentially adaptive mutations. See the Python documentation or the tables below for further details on these functions. Genomes of large population samples can be written to a file in a compressed format using the function

```
hivpopulation.write_genotypes_compressed(filename, number_of_individuals)
```

where `filename` is the name of the file, in which the data are to be stored, and `number_of_individuals` if the size of the random sample. The data can be accessed again by the standard Numpy `load` function.

IV. PYTHON FUNCTIONS

A. haploid_lowd

Definition	Description
`__init__(L, rng_seed)`	constructor. `rng_seed` a seed for the random number generator
`L`	number of loci
`N`	population size
`generation`	current generation (starts at zero)
`carrying_capacity`	carrying capacity of the environment, or target population size
`circular`	switch for circular or linear genomes (`True/False`)
`outcrossing_rate`	probability of sexual reproduction per generation
`recombination_model`	model of recombination: `FREE_RECOMBINATION`, `SINGLE_CROSSOVER` or `CROSSOVERS`
`status()`	prints a status list of the population parameters
`set_wildtype(N)`	initializes population of N individuals with the minus allele at all loci
`set_allele_frequencies(frequencies, N)`	initializes population in linkage equilibrium with specified allele frequencies
`set_genotypes(genotypes, counts)`	initializes population with fixed counts for specific genotypes. `genotypes` is a list of integers between 0 and $2^L - 1$.
`set_fitness_additive(coefficient)`	sets an additive fitness landscape
`set_fitness_function(genotypes, values)`	sets the fitness landscape for individual genotypes
`set_recombination_rates(rates, model=None)`	sets the recombination rate(s). `rates` can be a single value or a list. `model` can be `FFPopSim.SINGLE_CROSSOVER` or `FFPopSim.CROSSOVERS`
`set_mutation_rates(rates, rates_back=None)`	sets the mutation rate(s). `rates` specifies the forward mutation rate(s) and is a single value or a list. `rates_back` speficies the backward rates
`get_mutation_rates(locus=None, direction=None)`	returns the mutation rate(s). `direction` is 0 for forward, 1 for backward rates. Defaults to all sites and both directions
`evolve(gen=1)`	evolves the population for `gen` generations
`evolve_deterministic(gen=1)`	evolves the population for `gen` generations, skipping the resampling
`evolve_norec(gen=1)`	evolves the population for `gen` generations without recombination
`random_genomes(n_sample)`	returns `n_sample` random genomes from the population
`get_allele_frequency(locus)`	returns allele frequency at specified locus
`get_allele_frequencies()`	returns all allele frequencies
`get_genotype_frequency(genotype)`	returns the population frequency of `genotype`, an integer between 0 and $2^L - 1$
`get_genotype_frequencies()`	returns all genotype frequencies
`get_pair_frequency(locus1, locus2)`	returns the joint frequency of both alleles
`get_chi(locus)`	returns $\chi_{\texttt{locus}}$, a measure of the allele frequency varying between -1 and 1
`get_LD(locus1, locus2)`	returns the linkage disequilibrium between two loci
`get_chi2(locus1, locus2)`	returns $\chi_{\texttt{locus1,locus2}} := 4 \cdot \mathrm{LD}_{\texttt{locus1,locus2}}$
`get_moment(locus1, locus2)`	returns $\langle s_{\texttt{locus1}} s_{\texttt{locus2}} \rangle$, where $s_i \in \{\pm 1\}$
`get_allele_entropy`	returns the allele entropy of the population
`get_genotype_entropy`	returns the genotype entropy of the population
`get_fitness(genotype)`	returns the fitness of a specified genotype
`get_fitnesses()`	returns the fitness of all possible genotypes
`get_fitness_statistics()`	returns the mean and variance of the fitness in the population
`get_fitness_histogram(n_sample=1000, **kwargs)`	returns a histogram of the fitness in the population
`plot_fitness_histogram(axis=None, n_sample=1000, **kwargs)`	returns a plot handle of the histogram of the fitness in the population. `axis` enables plotting in an extant axis
`get_divergence_statistics(n_sample=1000)`	returns the mean and variance of divergence from wildtype in the population
`get_divergence_histogram(bins=10, n_sample=1000, **kwargs)`	returns a histogram of divergence in the population
`plot_divergence_histogram(axis=None, n_sample=1000, **kwargs)`	returns a plot handle of the histogram of divergence in the population. `axis` enables plotting in an extant axis
`get_diversity_statistics(n_sample=1000)`	returns the mean and variance of diversity in the population
`get_diversity_histogram(bins=10, n_sample=1000, **kwargs)`	returns a histogram of diversity in the population
`plot_diversity_histogram(axis=None, n_sample=1000, **kwargs)`	returns a plot handle of the histogram of diversity in the population. `axis` enables plotting in an extant axis

B. haploid_highd

Definition	Description
`__init__(L, rng_seed, number_of_traits=1)`	constructor. `rng_seed` a seed for the random number generator
`L`	number of loci
`N`	population size
`number_of_traits`	number of phenotypic traits
`generation`	current generation (starts at zero)
`participation_ratio`	participation ratio
`number_of_clones`	number of genetic clones
`carrying_capacity`	carrying capacity of the environment, or target population size
`circular`	switch for circular or linear genomes
`outcrossing_rate`	probability of sexual reproduction per generation
`crossover_rate`	crossover rate per site per generation
`recombination_model`	model of recombination
`mutation_rate`	mutation rate per site per generation
`trait_weights`	weights of traits on fitness (by default only the first trait counts, i.e. $[1,0,\ldots,0]$)
`status()`	prints a status list of the population parameters
`set_wildtype(N)`	initializes population of N individuals with the minus allele at all loci
`set_allele_frequencies(frequencies, N)`	initializes population in linkage equilibrium with specified allele frequencies
`set_genotypes(genotypes, counts)`	initializes population with fixed counts for specific genotypes. `genotypes` is a list, each element (genotype) being a Boolean list, e.g. `genotypes[0] = [0,1,1,0]`
`add_genotype(genotype, n=1)`	adds `genotype` (a Boolean list) to the population in `n` copies
`set_trait_additive(coefficients, t=0)`	sets an additive landscape for the `t`-th trait
`add_trait_coefficient(value, loci, t=0)`	sets $t_{\texttt{loci}}$, epistatic coefficient for the `t`-th trait. E.g. if `loci = [1,3]`, set t_{13}
`set_random_trait_epistasis(std, t=0)`	sets random epistatic coefficients with standard deviation `std` for the `t`-th trait
`clear_trait(t=0)`	clears landscape for the `t`-th trait
`clear_traits()`	clears all trait landscapes
`evolve(gen=1)`	evolves the population for `gen` generations
`bottleneck(size_of_bottleneck)`	makes the population undergo a bottleneck
`flip_single_locus(locus)`	creates a new clone by flipping `locus` in a random clone
`calc_stat()`	calculates trait and fitness statistics for the population
`random_clone()`	returns a random clone index from the population
`random_clones(n_sample)`	returns `n_sample` random clone indices from the population
`random_genomes(n_sample)`	returns `n_sample` random genomes from the population
`get_clone_size(n)`	returns size of the `n`-th clone
`get_clone_sizes()`	returns size of all clones
`get_genotype(n)`	returns the genotype (Boolean list) of the `n`-th clone
`get_genotypes(ind)`	returns an array of genotypes for clone indices in `ind`. Defaults to all genotypes
`get_allele_frequency(locus)`	returns allele frequency at specified locus
`get_allele_frequencies()`	returns all allele frequencies
`get_pair_frequency(locus1, locus2)`	returns the joint frequency of both alleles
`get_chi(locus)`	returns $\chi_{\texttt{locus}}$, a measure of the allele frequency varying between -1 and 1
`get_LD(locus1, locus2)`	returns the linkage disequilibrium between two loci
`get_chi2(locus1, locus2)`	returns $\chi_{\texttt{locus1,locus2}} := 4 \cdot \mathrm{LD}_{\texttt{locus1,locus2}}$
`get_moment(locus1, locus2)`	returns $\langle s_{\texttt{locus1}} s_{\texttt{locus2}} \rangle$, where $s_i \in \{\pm 1\}$
`get_trait_additive(t=0)`	get the additive coefficients of a phenotypic trait
`get_trait_statistics(t=0)`	get the mean and variance of a trait in the population
`get_trait_covariance(t1, t2)`	get the covariance of two traits in the population
`get_fitness(n)`	returns the fitness of the `n`-th clone
`get_fitnesses()`	returns the fitness of all possible genotypes
`get_fitness_statistics()`	returns the mean and variance of the fitness in the population
`get_fitness_histogram(bins=10, n_sample=1000,**kwargs)`	returns a histogram of the fitness in the population
`plot_fitness_histogram(axis=None, n_sample=1000, **kwargs)`	returns a plot handle of the histogram of the fitness in the population. `axis` enables plotting in an extant axis

Continued on next page

Definition	Description
get_divergence_statistics(n_sample=1000)	returns the mean and variance of divergence from wildtype in the population
get_divergence_histogram(bins=10, chunks=None, every=1, n_sample=1000, **kwargs)	returns a histogram of divergence in the population. chunks is a list of pairs, each for a genomic region of interest; every enables comparisons of subsets, e.g. only at third-codon positions (every=3)
plot_divergence_histogram(axis=None, n_sample=1000, **kwargs)	returns a plot handle of the histogram of divergence in the population. axis enables plotting in an extant axis
get_diversity_statistics(n_sample=1000)	returns the mean and variance of diversity in the population
get_diversity_histogram(bins=10, chunks=None, every=1, n_sample=1000, **kwargs)	returns a histogram of diversity in the population. Arguments as in get_divergence_histogram
plot_diversity_histogram(axis=None, n_sample=1000, **kwargs)	returns a plot handle of the histogram of diversity in the population. axis enables plotting in an extant axis

C. hivpopulation

As a subclass of haploid_highd, this class inherits its methods. Only different or new methods are listed.

Definition	Description
__init__(N=0, rng_seed=0, mutation_rate=3e-5, coinfection_rate=0.01, crossover_rate=0.001)	constructor. rng_seed a seed for the random number generator. A wildtype population of size N is generated. If N is zero, you have to initialize the population explicitely before performing any simulations
treatment	treatment weight (between 0 and 1). See also trait_weights of haploid_highd.
set_trait_landscape(traitnumber=0, lethal_fraction=0.05, deleterious_fraction=0.8, adaptive_fraction=0.01, effect_size_lethal=0.8, effect_size_deleterious=0.1, effect_size_adaptive=0.01, env_fraction=0.1, effect_size_env=0.01, number_epitopes=0, epitope_strength=0.05, number_valleys=0, valley_strength=0.1)	sets a random landscape for a phenotypic trait with certain statistical properties. env_fraction is the fraction of adaptive sites in the env gene, effect_size_env their average selection coefficient. Epitopes are short genomic regions of negative epistasis, valleys are regions of positive epistasis.
set_replication_landscape(**kwargs)	sets a random landscape for the replication capacity. It calls set_trait_landscape internally, with traitnumber=0
set_resistance_landscape(**kwargs)	sets a random landscape for the drug resistance. It calls set_trait_landscape internally, with traitnumber=1
set_replication_additive(coefficients)	sets an additive landscape for the replication capacity
set_resistance_additive(coefficients)	sets an additive landscape for the drug resistance
get_replication_additive()	returns the additive coefficients of the replication capacity
get_resistance_additive()	returns the additive coefficients of the drug resistance
read_replication_coefficients(filename)	reads additive replication coefficients from a text file
read_resistance_coefficients(filename)	reads additive resistance coefficients from a text file
write_genotypes(filename, sample_size, gt_label='', start=0, length=0)	stores sample_size random genotypes in a plain text file, in FASTA format. gt_label is a common label of those sequences, start is the position in the HIV genome to start storing, length the length of the region to store
write_genotypes_compressed(filename, sample_size, gt_label='', start=0, length=0)	stores sample_size random genotypes in a compressed file. Arguments have the same meaning as in write_genotypes. The sequences can be extracted again via the numpy.load function

Note: In histogram functions, **kwargs are passed to numpy.histogram; in plot functions, they are passed to matplotlib.pyplot.hist. In hivpopulation.set_replication_landscape and hivpopulation.set_resistance_landscape, the keyword arguments are the same as for hivpopulation.set_trait_landscape.

V. EXAMPLES

In the following, we list examples of the usage of FFPopSim for a small number of loci (`haploid_lowd`) and many loci (`haploid_highd` and `hivpopulation`). Additional examples are provided in the online documentation.

A. `haploid_lowd`: Valley crossing

One of the most striking effects of genetic epistasis is the slowdown of evolution when a combination of mutations is beneficial, but intermediate mutants are deleterious compared to wild type. Such scenario is relevant in applications, for instance for the emergence of bacterial or viral resistance to drugs (Weinreich *et al.*, 2005). Not surprisingly, recombination plays a central role in this process. On the one hand, it enhances the production rate of multiple mutants, on the other it depletes the class of complete mutants by back-recombination with deleterious backgrounds.

FFPopSim makes the efficient simulation of such processes as easy as the following script:

```python
import FFPopSim
L = 4                # number of loci
N = 1e10             # population size
s1 = 1e-5            # fitness of wild type (relative to intermediates)
s2 = 0.01            # fitness of quadruple mutant (relative to wild type)

pop = FFPopSim.haploid_lowd(L)         # create population
pop.set_genotypes([0b0], [N])          # start with N wild type individuals

pop.set_recombination_rates(1e-2)     # set recombination rate
pop.set_mutation_rates(1e-5)          # set mutation rate

# assign positive relative fitness to wild type and quadruple mutant
pop.set_fitness_function([0b0, 0b1111],
                         [s1, s1 + s2])

# cross valley with an accuracy of 100 generations
generations_at_once = 100
while (pop.get_genotype_frequency(0b1111) < 0.5) and (pop.generation < 1e7):
    pop.evolve(generations_at_once)

# print result
print 'Time to cross the valley: '+str(pop.generation)+' generations'
```

If the script is run with different recombination rates, the effect of this parameter on the time for valley crossing can be investigated, as shown in Fig. 2. The full script producing the figure is provided as separate file in the examples folder or online.

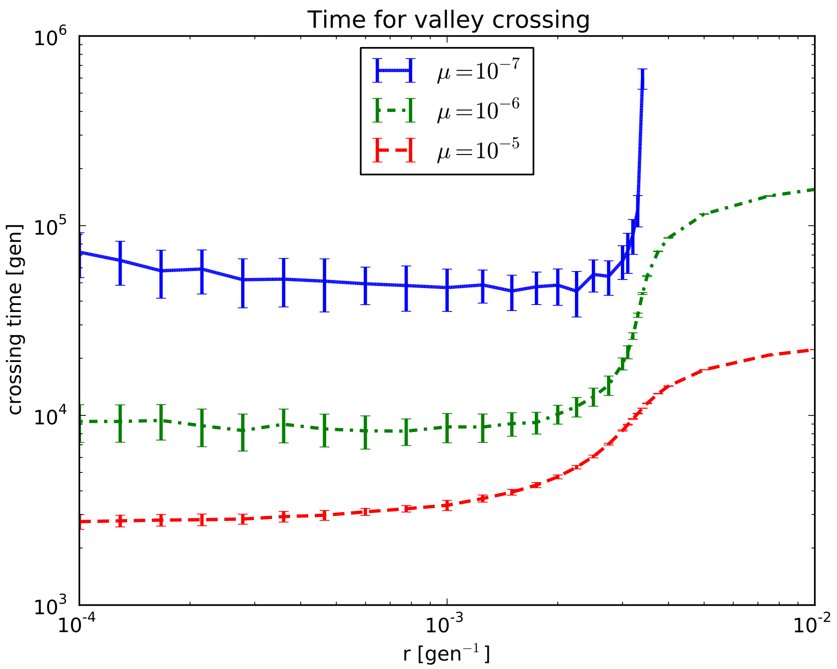

Figure 2 Example of the Python wrapper for `haploid_lowd`. The average time for crossing a 4 dimensional fitness valley is plotted against the recombination rate. Bars indicate the standard deviation across fifty repetitions. Crossing a valley becomes essentially impossible once the total recombination rate exceeds the fitness benefit, at least when mutation rates are small.

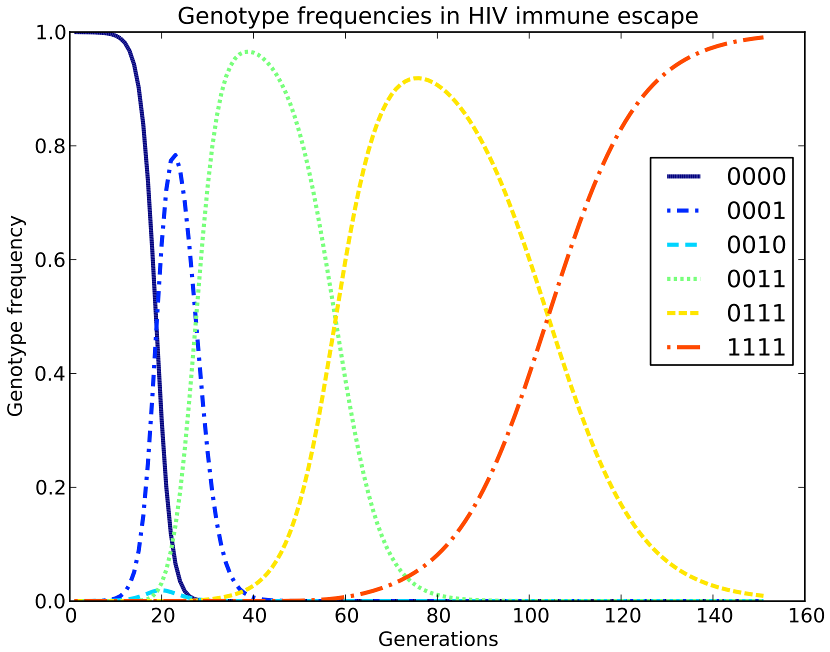

Figure 3 Example of the Python wrapper for `haploid_lowd`. The frequencies of some genotypes in the population during strong, parallel positive selection are shown.

B. haploid_lowd: HIV immune escape

During an HIV infection, the host immune system targets several viral epitopes simultaneously via a diverse arsenal of cytotoxic T-cells (CTLs). Mutations at several loci are thus selected for and start to rise in frequency at the same time. Because of the limited amount of recombination, different escape mutations compete against each other. This interference delays CTL escape.

The theoretical description of genetic interference is involved and often limited to two-loci models, but FFPopSim makes the simulation of this process straightforward. The following script evolves a four-loci population under parallel positive selection and tracks its genotype frequencies:

```
import FFPopSim
L = 4            # number of loci
N = 1e10         # population size

pop = FFPopSim.haploid_lowd(L)      # create population
pop.set_wildtype(N)                 # start with N wild type individuals

pop.set_recombination_rates(1e-4)   # set recombination rate
pop.set_mutation_rates(1e-5)        # set mutation rate

# set an additive fitness landscape (note that these coefficients are in +/- basis.
# Standard selection coefficents are twice as large)
pop.set_fitness_additive([0.3, 0.2, 0.1, 0.05])

# evolve until fixation of the quadruple mutant,
# storing times and genotype frequencies
times = []
genotype_frequencies = []
while (pop.get_genotype_frequency(0b1111) < 0.99) and (pop.generation < 1e7):
    pop.evolve()
    times.append(pop.generation)
    genotype_frequencies.append(pop.get_genotype_frequencies())
```

The resulting genotype frequencies are shown in Fig. 3. The full script producing the figure is provided as separate file in the examples folder or online.

C. haploid_highd: Genetic draft and drift

Neutral polymorphisms are strongly affected by linked selection. To demonstrate this effect, we simulate a large population of $N = 50000$ individuals and a genome of $L = 256$ loci. The majority of these loci are designated to be neutral and segregate at frequency 0.5. Mutations at every tenth locus have strongly deleterious effects and they hence are at a frequency close to zero. If once in a while one of these deleterious loci becomes beneficial due to an environmental change, new mutations will now rapidly sweep to fixation and perturb linked neutral diversity. This effect of genetic draft can be easily studied using haploid_highd. The following script produces the desired output.

```
import numpy as np
import FFPopSim as h

L=256   #simulate 256 loci

### set up
pop = h.haploid_highd(L)                         #produce an instance of haploid_highd with L loci
pop.carrying_capacity = 50000                    #set the average population size to 50000
pop.outcrossing_rate = 1                         #make the species obligate outcrossing
pop.crossover_rate = 0.02/pop.L                  #set the crossover rate to 0.02/L
pop.mutation_rate = 0.1/pop.carrying_capacity    #per locus mutation rate equal to 0.1/N

m=10
selection_coefficients = np.zeros(pop.L)         #most loci are neutral
selection_coefficients[::m] = -0.1               #every m-th locus is strongly deleterious

initial_allele_frequencies = 0.5*np.ones(pop.L)  #initial allele frequencies = 1/2
```

```python
initial_allele_frequencies[::m] = 0.0          #deleterious loci at frequency 0

# initialize the population in linkage equilibrium with the specified allele frequencies
pop.set_allele_frequencies(initial_allele_frequencies, pop.carrying_capacity)
pop.set_trait_additive(selection_coefficients)  #trait 0 is by default fitness

# keep track of allele frequencies
allele_frequencies = [pop.get_allele_frequencies()]
while pop.generation <2000:                       #evolve for at least 2000 generations
    pop.evolve(10)                                #procede 10 generations
    pop.calc_stat()
    allele_frequencies.append(pop.get_allele_frequencies())
    #every 200 generations, make one of the deleterious mutations beneficial
    if (pop.generation%200==0):
        selection_coefficients[m*np.random.randint(0,25)] = 0.01
        pop.set_trait_additive(selection_coefficients)      #update fitness function
```

The output of this script (amended by plotting commands) is shown in Fig. 4. The full script producing the figure is provides as separate file in the examples folder or online.

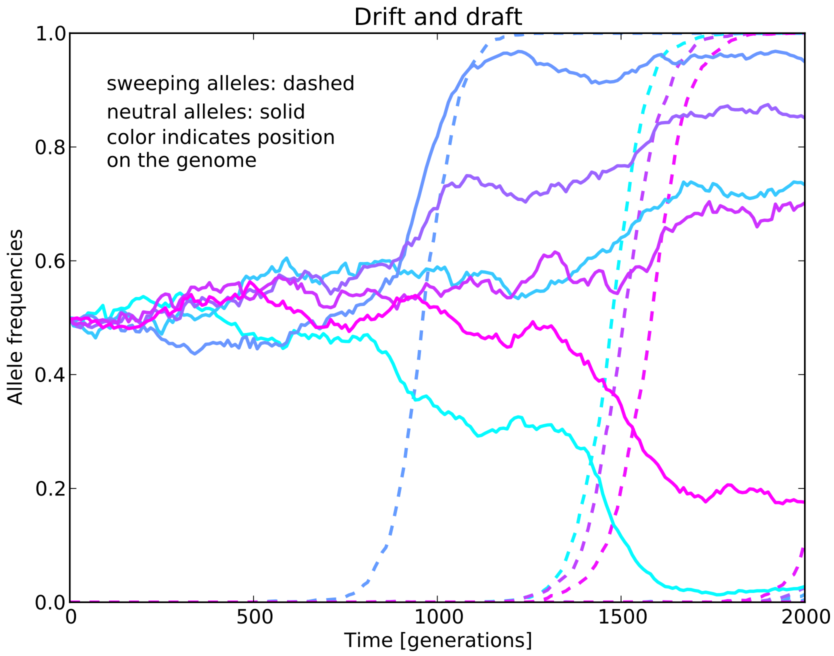

Figure 4 Example of the Python wrapper for `haploid_highd`. The frequencies of neutral alleles (solid lines) are strongly perturbed by tightly linked selective sweeps (dashed lines).

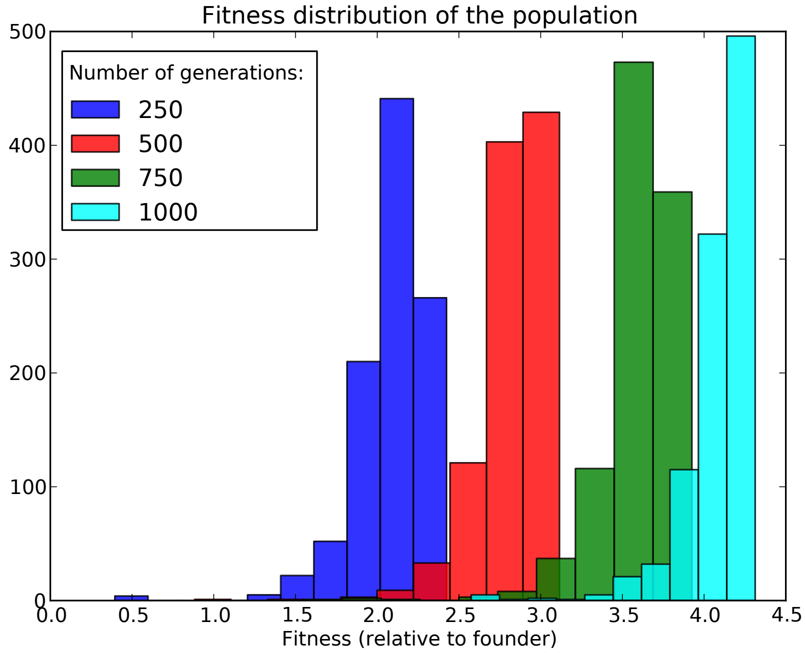

Figure 5 Example of the Python wrapper for `hivpopulation`. The fitness distribution of the population after 250, 500, 750, and 1000 generations from transmission is shown.

D. hivpopulation: HIV chronic infection

HIV evolution during chronic infection is determined by a number of parallel processes, such as mutation, recombination, and selection imposed by the immune system. In combination, these processes give rise to a complicated dynamics and don't understand how population features such as population diversity depend on model parameters. Hence simulations are an important source of insight.

FFPopSim offers a specific double C++/Python interface to this problem via its class `hivpopulation`. The following script simulates an HIV population for one thousand generations, under a random static fitness landscape, and stores a hundred random genomes every 250 generations in a compressed file:

```
import FFPopSim
N = 1000            # population size

# create the population.
# Default options:
# - wild type only
# - no treatment, i.e. replication capacity is the same as fitness
pop = FFPopSim.hivpopulation(N)

# set a random, additive replication capacity landscape
pop.set_replication_landscape(adaptive_fraction=0.01,
                              effect_size_adaptive=0.03)

# evolve the HIV population with an accuracy of 250 generations and
# store genomes in compressed format for further analysis
while pop.generation + 1 < 1000:
    pop.evolve(250)
    pop.write_genotypes_compressed('HIV_genomes'+str(pop.generation)+'.npz', 100)
```

NumPy can be used subsequently to analyze the genome sequences. Alternatively, the internal Python functions can be used, e.g. for calculating the fitness distribution directly using `hivpopulation.get_fitness_histogram`, as shown in Fig. 5. The full script producing the figure is provided as separate file in the examples folder or online.

References

Beazley, D. M., 2003, Future Gener. Comput. Syst. **19**(5), 599, ISSN 0167-739X, URL http://dx.doi.org/10.1016/S0167-739X(02)00171-1.

Hunter, J. D., 2007, Computing in Science and Engineering **9**(3), 90, ISSN 1521-9615, URL http://dx.doi.org/10.1109/MCSE.2007.55.

Neher, R., and B. Shraiman, 2011, Rev. Mod. Phys. **83**(4), 1283.

Oliphant, T., 2006, *A Guide to NumPy*, volume 1 (Trelgol Publishing).

Weinberger, E., 1991, Biological Cybernetics **65**, 321.

Weinreich, D. M., R. A. Watson, and L. Chao, 2005, Evolution **59**(6), 1165.
